# CRISTAL (a cluster-randomised, crossover, non-inferiority trial of aspirin compared to low molecular weight heparin for venous thromboembolism prophylaxis in hip or knee arthroplasty, a registry nested study): statistical analysis plan

**DOI:** 10.1186/s13063-021-05486-0

**Published:** 2021-08-24

**Authors:** Verinder Singh Sidhu, Thu-Lan Kelly, Nicole Pratt, Steven Graves, Rachelle Buchbinder, Justine Naylor, Richard de Steiger, Ilana Ackerman, Sam Adie, Michelle Lorimer, Durga Bastiras, Kara Cashman, Ian Harris

**Affiliations:** 1 Whitlam Orthopaedic Research Centre, Ingham Institute for Applied Medical Research, South West Sydney Clinical School, The University of New South Wales Sydney, Sydney, NSW Australia; 2 Clinical and Health Sciences, Quality Use of Medicines Pharmacy Research Centre, University of South Australia, Adelaide, Australia; 3 Australian Orthopaedic Association National Joint Replacement Registry, Adelaide, South Australia Australia; 4 Department of Epidemiology and Preventive Medicine, School of Public Health & Preventive Medicine, Monash University, Melbourne, Victoria Australia; 5 Monash Department of Clinical Epidemiology, Cabrini Institute, Melbourne, Victoria Australia; 6 Department of Surgery, Epworth Healthcare, University of Melbourne, Melbourne, Victoria Australia; 7 St. George and Sutherland Clinical School, The University of New South Wales Sydney, Sydney, NSW Australia; 8 South Australian Health and Medical Research Institute, Adelaide, South Australia Australia; 9 Institute of Musculoskeletal Health, School of Public Health, The University of Sydney, Sydney, NSW Australia

**Keywords:** Venous thromboembolism, Hip arthroplasty, Knee arthroplasty, Aspirin, Low molecular weight heparin, Statistical analysis plan

## Abstract

**Background:**

This a priori statistical analysis plan describes the analysis for CRISTAL.

**Methods:**

CRISTAL (cluster-randomised, crossover, non-inferiority trial of aspirin compared to low molecular weight heparin for venous thromboembolism prophylaxis in hip or knee arthroplasty, a registry nested study) aims to determine whether aspirin is non-inferior to low molecular weight heparin (LMWH) in preventing symptomatic venous thromboembolism (VTE) following hip arthroplasty (HA) or knee arthroplasty (KA). The study is nested within the Australian Orthopaedic Association National Joint Replacement Registry. The trial was commenced in April 2019 and after an unplanned interim analysis, recruitment was stopped (December 2020), as the stopping rule was met for the primary outcome.

The clusters comprised hospitals performing > 250 HA and/or KA procedures per annum, whereby all adults (> 18 years) undergoing HA or KA were recruited. Each hospital was randomised to commence with aspirin, orally, 85–150 mg daily or LMWH (enoxaparin), 40 mg, subcutaneously, daily within 24 h postoperatively, for 35 days after HA and 14 days after KA. Crossover was planned once the registration target was met for the first arm.

The primary end point is symptomatic VTE within 90 days. Secondary outcomes include readmission, reoperation, major bleeding and death within 90 days, and reoperation and patient-reported pain, function and health status at 6 months.

The main analyses will focus on the primary and secondary outcomes for patients undergoing elective primary total HA and KA for osteoarthritis. The analysis will use an intention-to-treat approach with cluster summary methods to compare treatment arms. As the trial stopped early, analyses will account for incomplete cluster crossover and unequal cluster sizes.

**Conclusions:**

This paper provides a detailed statistical analysis plan for CRISTAL.

**Trial registration:**

Australian and New Zealand Clinical Trials Registry ACTRN12618001879257. Registered on 19/11/2018.

## Background

Despite the increasing use of aspirin as a sole chemotherapeutic agent for symptomatic venous thromboembolic event (VTE) prophylaxis following hip arthroplasty (HA) and knee arthroplasty (KA) [1], there remains limited high-quality comparative evidence for its safety and efficacy. The majority of studies supporting the safety and efficacy of aspirin compared to other agents, including low molecular weight heparin (LMWH), have been retrospective or non-randomised [2–11]. The only randomised trials have been underpowered or have used an alternative form of prophylaxis (e.g., LMWH or a novel oral anticoagulant (NOAC)) for the immediate postoperative period following HA or KA prior to changing to aspirin for extended prophylaxis, which does not reflect the way aspirin is used in Australia [12, 13]. CRISTAL is a pragmatic, multicentre cluster-randomised, two-period cross-sectional crossover trial that aims to determine if aspirin is non-inferior to LMWH in the prevention of symptomatic VTE following HA and KA. It is nested within the Australian Orthopaedic Association National Joint Replacement Registry (AOANJRR).

The trial commenced in April 2019 and the estimated timeline for completion of patient registration was 24 months. However, after an unplanned interim analysis in which the trial stopping rule was met, patient registration was ceased in December 2020, resulting in incomplete crossover. This statistical analysis plan details the planned analyses for CRISTAL to facilitate transparency of data analysis. The CONSORT statement for cluster randomised trials was referred to in preparation of this document [14]. The trial protocol has previously been published [15].

## Study overview

### Ethics

Ethics approval was granted from all relevant central, lead ethics committees involved and all participating hospitals, as outlined in the published trial protocol [15]. The trial is registered with the Australian New Zealand Clinical Trials Registry (ACTRN12618001879257p) and is endorsed by the Australia and New Zealand Musculoskeletal (ANZMUSC) Clinical Trials Network.

### Participating hospitals and patient registration

The clusters in CRISTAL were defined as hospitals where hip and knee arthroplasty procedures were performed. Hospitals were eligible for recruitment provided they agreed to follow the trial protocol and if they performed greater than 250 HA and/or KA procedures per annum. There were 31 hospitals (clusters) that were recruited.

Each recruited hospital was responsible for registering patients and complying with the trial protocol. The AOANJRR routinely collects data pertaining to the procedure, patient age, sex, American Society of Anaesthesiologists (ASA) class and body mass index (BMI) and death on all patients undergoing HA and KA procedures. Patient-reported outcomes are collected through the electronic Clinical Trials Platform, which requires pre-operative registration of the patient onto the electronic system. All adult (age 18 and older) patients undergoing HA or KA were eligible for registration into the study and eligible to receive the allocated study drug, except for those who were already on long-term anticoagulation (specifically a NOAC, warfarin or dual antiplatelet therapy (DAPT)) and those with a medical contraindication to either drug, e.g., an allergy or a medical comorbidity such as thrombophilia that precluded treatment with the study drug.

Patients who were not registered in the electronic Clinical Trials Platform will be included in secondary analyses, as procedure information, demographics and mortality were still recorded even though the primary outcome and other patient-reported outcomes were not recorded.

### Intervention

Each hospital (cluster) was allocated to consecutive periods of a standard protocol of LMWH and a standard protocol of aspirin as VTE prophylaxis, with the order being randomised. Patients in the aspirin group received aspirin at 85–150 mg once daily, orally for 35 days post HA and for 14 days post KA, commencing within 24 h of surgery. Patients in the LMWH group received enoxaparin at 40 mg once daily, subcutaneously for the same time periods, with this dose reduced to 20 mg for patients who weigh less than 50 kg and for patients with an estimated glomerular filtration rate (eGFR) of less than 30 mL/min who are not on dialysis. Other interventions that were standard across all sites were the intra- and post-operative use of intermittent pneumatic compression (IPC) calf devices until patients are mobile, the use of compression stockings, and mobilisation offered on day 0 or day 1 postoperatively.

### Randomisation and allocation

Study investigators have remained blinded to group allocation. All 31 participating hospitals were randomised to commence with either LMWH or aspirin, in randomly permuted blocks of size four by statisticians from the South Australian Health and Medical Research Institute (SAHMRI), independent of study investigators. The randomisation sequence was generated using an online application (https://www.sealedenvelope.com) and this was provided to an unblinded data manager from SAHMRI. The hospital was then allocated to a treatment sequence by SAHMRI staff and this information was provided to the AOANJRR (independent of study investigators), with the site being informed of their allocated treatment arm the week prior to commencing initial patient registration. Hospitals followed the designed protocol for patients for their allocated treatment arm and were advised to crossover to the alternate treatment once the sample size for the first treatment arm was met.

For clusters that did not reach the sample size for the first arm within 18 months of commencement, crossover occurred prior to reaching the sample size so that an equal number of patients could be registered in each arm within the study timeframe.

### Evaluation of adherence to the study protocol and protocol deviations

At a hospital level, during the course of the trial, each hospital was audited within the first month of each treatment arm to ensure they were complying with the trial protocol and to ensure each cluster received the intended allocated treatment. The audit consisted of the first 20 patients of each treatment arm. If a site had a compliance of less than 80%, the site was educated on methods of improving protocol compliance and subsequently re-audited until compliance to the protocol was above 80%.

Hospitals were also advised to inform trial co-ordinators of patients not receiving the correct study drug or those patients who had the study drug withheld for greater than 48 hours due to side effects (e.g. allergy, excessive wound drainage or bleeding events). These protocol deviations were recorded using the Clinical Trials Platform.

### Outcome variables

The primary outcome of the study is symptomatic VTE within 90 days of surgery. Secondary outcomes are as follows:

- Deep vein thrombosis (DVT) only (total, below-knee and above-knee) within 90 days
- Pulmonary embolism (PE) only within 90 days
- Readmission related to the original surgery or associated treatment (including bleeding and VTE-related) within 90 days
- Reoperation on the same joint within 90 days and within 6 months of surgery
- Major bleeding events within 90 days defined as bleeding events resulting in readmission, reoperation or death
- Death within 90 days
- Change in patient-reported pain, function and health status measures as measured by the Oxford Hip Score (OHS), Oxford Knee Score (OKS), EQ-5D score, and the EQ-VAS from baseline to 6 months postoperatively

Outcome and demographic data were collected preoperatively (demographics, patient-reported pain, function and health status) and at 90 days and 6 months postoperatively. Data for all primary and secondary outcomes are patient-reported (except for death). All patients who responded ‘yes’ to having experienced a VTE or a secondary operation within 6 months had this result verified by AOANJRR staff through contact with treating doctors and hospitals. A random audit of 200 patients who did not report a VTE event was undertaken to detect the false negative reporting rate. All data collected for registered patients specific to CRISTAL have been outlined in the published protocol [15]. Mortality data were collected through linkage between the AOANJRR and the National Death Index.

In the published protocol [15], mortality was to be measured at 90 days and 6 months. Due to the lack of sensitivity in measuring VTE-related mortality at 6 months, and due to the lag in data availability for mortality, we will only analyse mortality at 90 days [16].

### Power and sample size

For the sample size calculation in CRISTAL, we used an estimated overall event rate of 2% (based on the current available literature) [17, 18], a non-inferiority margin of 1% (based on clinician opinion and a recent randomised controlled trial) [12], i.e., an event rate of 2.5% for aspirin and 1.5% for LMWH, a power of 90% and a one-sided significance level of 0.025. For an individual randomised trial, this yields a sample size of 4,117 per treatment group or a total of 8,234 patients. For a cluster-randomised crossover trial with an intracluster correlation of 0.01, an interperiod correlation of 0.008 and 31 clusters, the required sample size is 11,160 patients, or 180 patients per arm for each cluster [19, 20]. However, due to the uncertainty surrounding the event rate and intracluster and interperiod correlations, loss to follow-up, uneven recruitment rates leading to unequal cluster sizes or clusters dropping out of the study, we aimed to register 251 patients eligible for the primary objective of the study, providing a total of 15,562 patients. This figure allowed for a maximum 27% reduction in the above sample size calculation [15]; however, the actual loss to follow-up was expected to be less than this.

## Statistical analysis plan

### Patient populations and subgroups for analyses

**Fig. 1:**
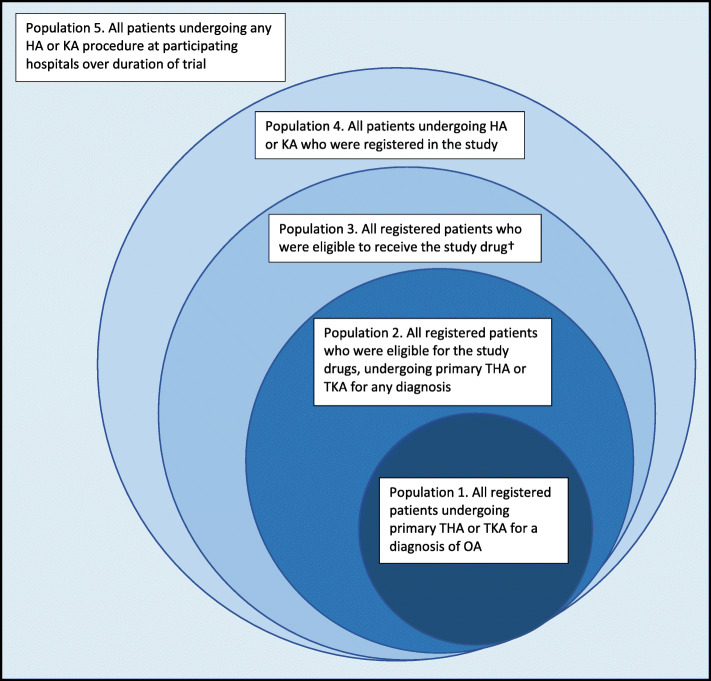
Patient populations within CRISTAL. Abbreviations: *HA* hip arthroplasty, *KA* knee arthroplasty, *THA* total hip arthroplasty, *TKA* total knee arthroplasty, *OA* osteoarthritis. Study drug excluded for patients who already on long-term anticoagulation (specifically a novel oral anticoagulant – NOAC, warfarin or dual antiplatelet therapy – DAPT) and those who have a medical contraindication

The total patient population for CRISTAL comprises all patients undergoing HA or KA at participating institutions over the duration of the study, regardless of whether these patients were registered or eligible to receive the study drug (defined as population 5, see Fig. 1).

Within this total population, the following populations will be used to form the basis of the analyses:

- Registered patients undergoing any form of HA or KA (including partial or revision surgery, for any indication) regardless of eligibility to receive the study drug (population 4)
- Registered patients undergoing any form of HA or KA (including partial or revision surgery, for any indication) who were eligible to receive the study drug (population 3)
- Registered patients undergoing elective primary THA or TKA (for any indication) who were eligible to receive the study drug (population 2)
- All registered patients undergoing elective primary THA or TKA for a recorded diagnosis of osteoarthritis (OA) who were eligible to receive the study drug (population 1)

These populations are represented diagrammatically in Fig. 1.

Each outcome (primary and secondary) will be assessed for populations 1, 2, 3 and 4 listed in Fig. 1. Mortality will be assessed for all populations (including population 5). The primary objective of the study as outlined in the published protocol [15], was the analysis of population 1 only (registered patients undergoing primary THA or TKA for a diagnosis of OA who are eligible to receive the study drug), as this was the focus of the sample size calculation. This population will remain the focus of the main analyses.

Population 1 was chosen as the focus of the main analysis as these patients represent the majority of patients undergoing HA or KA procedures and there are known differences in outcomes and co-morbidities with other diagnoses (e.g. fracture, tumour), which could confound the primary outcome [21].

For the primary end point of VTE, the following subgroup analyses will be conducted within the corresponding populations listed:

- Type of joint replacement: primary THA compared to primary TKA—population 1
- Bilateral arthroplasty: patients undergoing simultaneous bilateral arthroplasty compared to those who are not—population 1
- Revision arthroplasty: patients undergoing revision hip or knee arthroplasty compared to those undergoing primary arthroplasty—population 3
- Prior history of VTE: patients with a prior history of VTE compared to those without—population 1

### Analysis principles

Data will be analysed according to the intention-to-treat principle with clusters analysed according to assigned group allocation. Although hospital and patient protocol deviations will be recorded, no as-treated analyses will be performed, as there are no verified data available to determine whether individual patients received the assigned study drug for the full period, given the pragmatic nature of the trial. The timing of analyses will be stratified by the follow-up time of the outcomes measured (90 days and 6 months). The difference in absolute risk for symptomatic VTE between each group and 95% confidence intervals (upper and lower) will be examined to determine if the non-inferiority margin is met.

Continuous variables will be summarised using standard measures of central tendency and dispersion, using either mean and standard deviation or median and interquartile range. Categorical variables will be summarised by frequencies and percentages.

Analyses will be performed using SAS version 9.4 (SAS Institute, Cary USA) and R (R Foundation for Statistical Computing Platform) version 4.0.2 or higher.

### Interim analysis

An interim analysis was not initially planned, as both treatments are considered standard practice for VTE prophylaxis in Australia and the trial is investigating an adverse event as the primary outcome. However, due to concerns of an increased adverse event rate (symptomatic VTE and death) in one of the prophylaxis groups, a Data Safety Monitoring Board (DSMB) was convened 1 year into patient recruitment. The DSMB consisted of an orthopaedic surgeon, a haematologist and a statistician, all independent of the trial.

The DSMB were advised by the Trial Management Committee (TMC) to conduct an interim analysis. In conjunction with the DSMB (prior to the interim analysis), the TMC applied the Haybittle-Peto stopping rule of a two-sided significance of 0.001 for the primary outcome in population 1 [22, 23]. This stopping rule was chosen as it does not require adjustment of the significance threshold for the final analysis and allows further interim analyses using the same threshold (if required).

After the first interim analysis (in September 2020), the DSMB recommended continuing the trial and performing a second interim analysis in November 2020. After reviewing the second interim analysis, the DSMB recommended ceasing patient recruitment as the stopping rule had been met. The study ceased recruiting patients in December 2020 and sites reverted to their usual VTE prophylaxis pathways.

### Methods used for interim analyses

Interim analyses were conducted for VTE and mortality within 90 days for population 1. To account for unequal cluster sizes, incomplete crossover or clusters which had not yet crossed over, a composite analysis was designed. For clusters which had crossed over, including with partial completion of the second period, the cluster weighted estimator intended for the primary outcome was used. Harmonic mean weighting when there are unequal cluster sizes has been shown to improve precision and 95% confidence interval coverage compared with unweighted or inverse variance estimates [24, 25]. Clusters which had not crossed over were analysed using the cluster period summaries, weighted by cluster size, in a parallel design approach, i.e., as if it were a cluster randomised trial without crossover. Estimates for the two approaches were combined using inverse variance weights to provide a final estimate. Confidence intervals were constructed using the Haybittle-Peto boundary of 0.001.

### Data integrity

Integrity of data will be checked prior to conducting the final analysis. The data set will be checked for errors, omissions and double data entry. These will be resolved prior to commencing the analysis in consultation with the data management plan [15].

### Blinding

The DSMB were blinded to treatment allocation (groups in the interim analyses were labelled A and B). All researchers involved in the preparation of this analysis plan will have no access to trial data broken down by treatment allocation for the final statistical analysis. Once data integrity checks have been conducted, a blind review to quantify missing data of the entire dataset will be conducted and any final amendments to the statistical analysis plan will be made before the database is locked. During analysis and interpretation, group allocation will be masked by dummy group names and the true allocation will be unmasked only after the final statistical report has been completed and interpretation has been agreed to by the writing group and minuted.

### Methods for handling missing data

Multiple imputation using chained equations will be used to account for missing data. The imputation model will use auxiliary variables gathered from routine AOANJRR data (including age, sex, baseline health, pain and function, diagnosis and surgical factors), as well as cluster and period effects. One hundred datasets will be imputed at the patient level, then each dataset will be analysed using the main analysis method with cluster summaries and combined using Rubin’s rules. If there is any possibility of bias due to perfect prediction of rare outcomes such as VTE [26] or imputing values out of range for bounded variables such as pain scores or EQ5D [27], multiple imputation using chained equations will not be performed. Since the most likely reason for loss to follow-up is difficulty in contacting patients postoperatively (rather than association with treatment assignment or outcome), missing data will be assumed to be missing at random.

As a further sensitivity analysis for the primary outcome only, inverse probability weighting, where the complete cases are weighted by the inverse probability of being the complete case will also be used to account for missing data. Inverse probability weighting has an advantage over multiple imputation when there are large blocks of missing data with either observed values for all variables or missing values for the majority of the variables, for example, pre-operative pain and function scores [28]. The inverse probability weights will be used to produce weighted cluster summaries, which will be analysed using the main analysis method, with cluster sizes calculated as the sum of the inverse probability weights.

### Trial profile and baseline characteristics

**Fig. 2:**
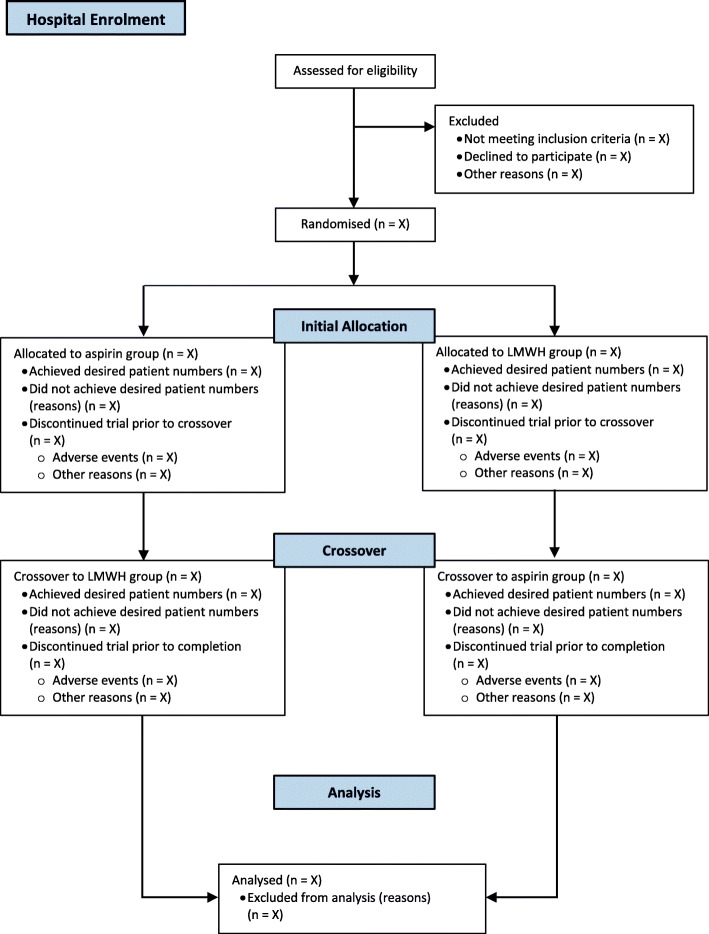
Flowsheet of participating hospitals

**Fig. 3:**
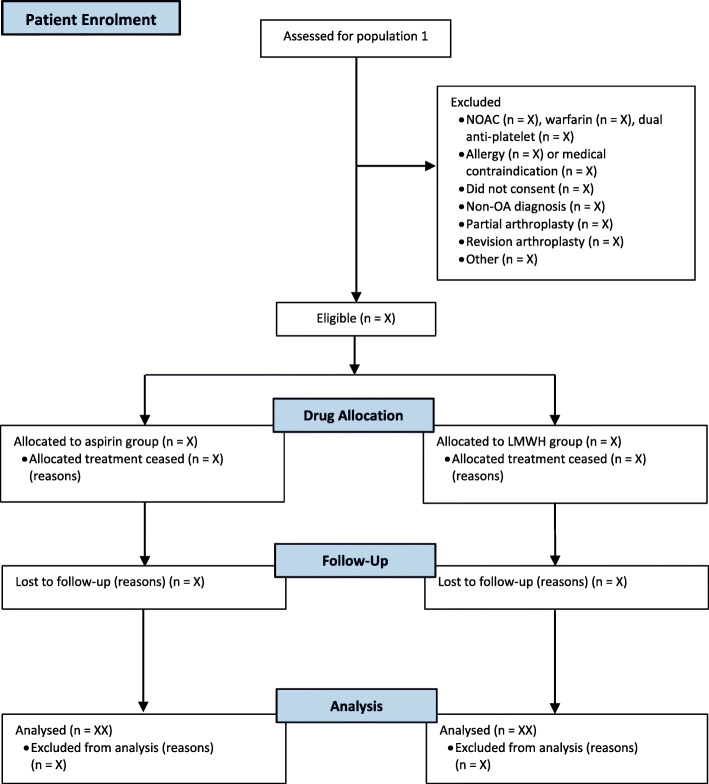
Flowsheet of patients within population 1. Population 1 refers to registered patients undergoing primary THA or TKA for a diagnosis of OA, who are eligible to receive the study drug

The flow of participating hospitals (including losses and exclusions) through the study and participating patients will be reported in line with the Consolidated Standards of Reporting Trials (CONSORT) statement (Figs. 2 and 3).

Baseline characteristics of participating clusters, including a number of annual HA and KA procedures performed in the year prior to trial commencement, hospital type (public or private hospital), initial treatment allocation and whether the hospital achieved crossover are shown in Table 1. This table also shows the number of patients registered for population 1 (the population used for the sample size calculation) by each participating hospital and the overall registration rate of each hospital will be presented as outlined. The overall registration rate describes the number of registered patients undergoing any HA or KA procedure (population 4) divided by the number of patients who underwent any HA or KA procedure over the duration of the trial at participating hospitals (regardless of whether they were registered – population 5). Hospital names will remain anonymous.

**Table 1:** Number of registered patients for population 1 by the treatment group and overall registration rate for each participating hospital

Hospital	Number of HA and KA procedures performed (2018)	Insurance status	Initial treatment allocation	Crossover achieved	LMWH group (population 1)	Aspirin group (population 1)	Overall registration rate (combined for both groups)
1					n	n	%
2					n	n	%
3					n	n	%
4					n	n	%
5					n	n	%
6					n	n	%
7					n	n	%
8					n	n	%
9					n	n	%
10					n	n	%
11					n	n	%
12					n	n	%
13					n	n	%
14					n	n	%
15					n	n	%
16					n	n	%
17					n	n	%
18					n	n	%
19					n	n	%
20					n	n	%
21					n	n	%
22					n	n	%
23					n	n	%
24					n	n	%
25					n	n	%
26					n	n	%
27					n	n	%
28					n	n	%
29					n	n	%
30					n	n	%
31					n	n	%
**Total**					**n**	**n**	**%**

Population 1 refers to registered patients undergoing primary THA or TKA for a diagnosis of OA, who are eligible to receive the study drug

Descriptive statistics of baseline patient characteristics for all registered patients eligible to receive the study drug (population 3) will be presented by the prophylaxis group (Table 2).

**Table 2:** Baseline patient characteristics for all registered patients eligible to receive study drug (population 3), according to treatment allocation

	LMWH (n = X)	Aspirin (n = X)
Age (years)	xx.x (xx.x – xx.x), n	xx.x (xx.x – xx.x), n
BMI (kg/m^2^)	xx.x (xx.x – xx.x), n	xx.x (xx.x – xx.x), n
Male sex	n (%)	n (%)
ASA grading		
1	n (%)	n (%)
2	n (%)	n (%)
3	n (%)	n (%)
4	n (%)	n (%)
5	n (%)	n (%)
Previous venous thromboembolism	n (%)	n (%)
Long term anticoagulant use		
Aspirin	n (%)	n (%)
Other single antiplatelet	n (%)	n (%)
Joint replacement		
THA	n (%)	n (%)
TKA	n (%)	n (%)
Other HA	n (%)	n (%)
Other KA	n (%)	n (%)
Bilateral	n (%)	n (%)
Type of surgery		
Primary total	n (%)	n (%)
Primary partial	n (%)	n (%)
Primary resurfacing	n (%)	n (%)
Revision	n (%)	n (%)
Other	n (%)	n (%)
Indication		
Osteoarthritis	n (%)	n (%)
Inflammatory	n (%)	n (%)
Avascular necrosis	n (%)	n (%)
Fracture	n (%)	n (%)
Other	n (%)	n (%)
Prosthesis		
Cemented	n (%)	n (%)
Hybrid	n (%)	n (%)
Uncemented	n (%)	n (%)
Pain and function		
Oxford Hip Score	xx.x (xx.x – xx.x), n	xx.x (xx.x – xx.x), n
Oxford Knee Score	xx.x (xx.x – xx.x), n	xx.x (xx.x – xx.x), n
EQ-5D	xx.x (xx.x – xx.x), n	xx.x (xx.x – xx.x), n
EQ-VAS	xx.x (xx.x – xx.x), n	xx.x (xx.x – xx.x), n

Abbreviations: *BMI* body mass index, *ASA* American Society of Anaesthesiologists

## Main analyses

The main analyses will include the primary and secondary outcomes for registered patients eligible to receive the study drug undergoing THA or TKA for a diagnosis of OA (population 1). In addition, the primary and secondary outcomes will be analysed for populations 2, 3 and 4. Mortality will also be analysed for population 5 (see ‘Additional analyses’ below).

**Fig. 4:**
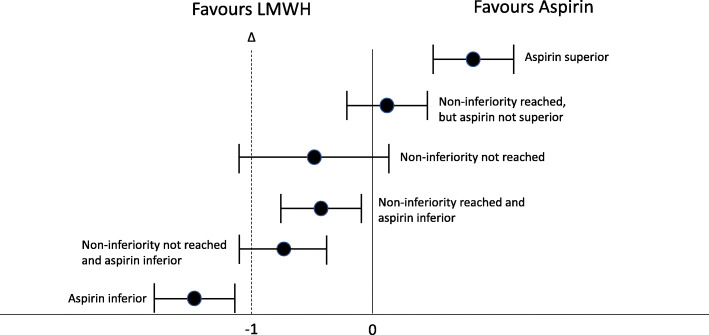
Between group change in overall 90-day symptomatic VTE rate and non-inferiority margin. The dotted line represents the non-inferiority margin

For the primary outcome, the analysis will test the between-group difference of cases developing a symptomatic VTE within 90 days for non-inferiority of aspirin at a margin of 1%. Cluster summary methods will be used to estimate the treatment effect using cluster level differences. These have been shown to be appropriate for cluster-randomised crossover trials with rare outcomes, and the intracluster and interperiod correlation coefficients expected in this trial. The crossover difference per cluster is the mean outcome for the intervention period minus the mean outcome for the control period. In a linear regression of cluster differences on treatment sequence, the treatment effect estimate is the intercept. To account for potential unequal cluster sizes, a cluster size weighted estimator will be used with harmonic mean weights of the number of patients in the two periods, which was the same method used in the interim analyses for incomplete crossover [24, 25]. Treatment effects will be presented as absolute risk differences and 95% confidence intervals will be examined to determine whether the non-inferiority margin has been met and whether the superiority of one drug can be concluded. The primary outcome will be presented in Table 3 and Fig. 4 will be used to demonstrate whether the non-inferiority margin has been met for population 1 [29].

**Table 3:** Outcomes for population 1

Outcome	LMWH allocation (n = X)	Aspirin allocation (n = X)	Absolute risk difference	95% confidence interval	***p*** value
**Any venous thromboembolism**	n (%)	n (%)	X	X – X	
**Type of venous thromboembolism**					
Pulmonary embolism	n (%)	n (%)	X	X – X	
Deep venous thrombosis	n (%)	n (%)	X	X – X	
Both Pulmonary embolism and deep venous thrombosis	n (%)	n (%)	X	X – X	
Above knee deep venous thrombosis	n (%)	n (%)	X	X – X	
Below knee deep venous thrombosis	n (%)	n (%)	X	X – X	
Death	n (%)	n (%)	X	X – X	
Re-operation (90 days)	n (%)	n (%)	X	X – X	
Reoperation (6 months)	n (%)	n (%)	X	X – X	
Re-admission	n (%)	n (%)	X	X – X	
Major bleeding	n (%)	n (%)	X	X – X	
Pain and function (median and IQR)^a^					
Oxford Hip Score	X (X – X)	X (X – X)	X	X – X	
Oxford Knee Score	X (X – X)	X (X – X)	X	X – X	
EQ-5D	X (X – X)	X (X – X)	X	X – X	
EQ-VAS	X (X – X)	X (X – X)	X	X – X	

^a^at 6 months

The secondary outcomes will investigate non-VTE complications (death, re-operation, readmission and major bleeding events) within 90 days, and reoperation and patient-related pain and function at 6 months (OHS, OKS, EQ-5D and EQ-VAS). Cluster summary methods will be used within an intention-to-treat approach. For binary outcomes, the cluster mean per period will be the proportion of patients who had the outcome, while for continuous outcomes such as pain score, the cluster mean will be the mean outcome. Treatment effects will be presented as absolute risk differences and 95% confidence intervals to determine if one treatment is superior to the alternative. Results for the secondary outcomes in population 1 will be presented in tabular form (Table 3) and results for the primary and secondary outcomes in populations 2, 3 and 4 in Table 4.

**Table 4:** Outcomes for populations 2, 3 and 4

Population	Outcome	LMWH allocation (*n* = X)	Aspirin allocation (*n* = X)	Absolute risk difference	95% confidence interval	***p*** value
All primary THA/TKA for any diagnosis eligible to receive study drug (population 2)	**Any venous thromboembolism**	n (%)	n (%)	X	X – X	
	**Type of venous thromboembolism**					
	Pulmonary embolism	n (%)	n (%)	X	X – X	
	Deep venous thrombosis	n (%)	n (%)	X	X – X	
	Both Pulmonary embolism and deep venous thrombosis	n (%)	n (%)	X	X – X	
	Above knee deep venous thrombosis	n (%)	n (%)	X	X – X	
	Below knee deep venous thrombosis	n (%)	n (%)	X	X – X	
	Death	n (%)	n (%)	X	X – X	
	Re-operation (90 days)	n (%)	n (%)	X	X – X	
	Reoperation (6 months)	n (%)	n (%)	X	X – X	
	Re-admission	n (%)	n (%)	X	X – X	
	Major Bleeding	n (%)	n (%)	X	X – X	
	Pain and Function (median and IQR)^a^					
	Oxford Hip Score	X (X – X)	X (X – X)	X	X – X	
	Oxford Knee Score	X (X – X)	X (X – X)	X	X – X	
	EQ-5D	X (X – X)	X (X – X)	X	X – X	
	EQ-VAS	X (X – X)	X (X – X)	X	X – X	
All HA/KA eligible to receive study drug (population 3)	**Any venous thromboembolism**	n (%)	n (%)	X	X – X	
	**Type of venous thromboembolism**					
	Pulmonary embolism	n (%)	n (%)	X	X – X	
	Deep venous thrombosis	n (%)	n (%)	X	X – X	
	Both Pulmonary embolism and deep venous thrombosis	n (%)	n (%)	X	X – X	
	Above knee deep venous thrombosis	n (%)	n (%)	X	X – X	
	Below knee deep venous thrombosis	n (%)	n (%)	X	X – X	
	Death	n (%)	n (%)	X	X – X	
	Re-operation (90 days)	n (%)	n (%)	X	X – X	
	Reoperation (6 months)	n (%)	n (%)	X	X – X	
	Re-admission	n (%)	n (%)	X	X – X	
	Major bleeding	n (%)	n (%)	X	X – X	
	Pain and Function (median and IQR)^a^					
	Oxford Hip Score	X (X – X)	X (X – X)	X	X – X	
	Oxford Knee Score	X (X – X)	X (X – X)	X	X – X	
	EQ-5D	X (X – X)	X (X – X)	X	X – X	
	EQ-VAS	X (X – X)	X (X – X)	X	X – X	
All HA/KA including study drug exclusion (population 4)	**Any venous thromboembolism**	n (%)	n (%)	X	X – X	
	**Type of venous thromboembolism**					
	Pulmonary embolism	n (%)	n (%)	X	X – X	
	Deep venous thrombosis	n (%)	n (%)	X	X – X	
	Both Pulmonary embolism and deep venous thrombosis	n (%)	n (%)	X	X – X	
	Above knee deep venous thrombosis	n (%)	n (%)	X	X – X	
	Below knee deep venous thrombosis	n (%)	n (%)	X	X – X	
	Death	n (%)	n (%)	X	X – X	
	Re-operation (90 days)	n (%)	n (%)	X	X – X	
	Reoperation (6 months)	n (%)	n (%)	X	X – X	
	Re-admission	n (%)	n (%)	X	X – X	
	Major bleeding	n (%)	n (%)	X	X – X	
	Pain and function (median and IQR)^a^					
	Oxford Hip Score	X (X – X)	X (X – X)	X	X – X	
	Oxford Knee Score	X (X – X)	X (X – X)	X	X – X	
	EQ-5D	X (X – X)	X (X – X)	X	X – X	
	EQ-VAS	X (X – X)	X (X – X)	X	X – X	

^a^at 6 months

Due to the early stopping of the trial, final analyses of the primary and secondary outcomes will use the same composite method as the interim analyses which accounts for clusters with incomplete as well as no crossover, with 95% confidence intervals. No bias is expected from early stopping if the patients included in the trial are not systematically different from later patients who would have been included after the trial was stopped. Our composite analysis method accounts for clusters which either had incomplete crossover and or did not crossover. However, the lower sample size and unequal cluster sizes decrease the precision of the outcome estimates. Since we used cluster weighted estimates to account for unequal cluster sizes and increased the initial sample size by 27% above the minimum required, the loss of precision will be mitigated. The trial was stopped based on the Haybittle-Peto boundary of 0.001, so we anticipate the final analysis using 95% confidence intervals will have sufficient power.

### Subgroup analyses

Subgroup analyses for the primary outcome (treatment group differences by subgroup) will include THA or TKA, bilateral or unilateral procedures, a prior history of VTE or not for population 1, and primary arthroplasty or revision arthroplasty for population 3.

To assess treatment effects for each subgroup separately, cluster summaries will be produced for each subgroup. An interaction term between treatment group and subgroup (e.g. THA/TKA, bilateral/unilateral) will be added to the model for the primary outcome. The treatment differences for each subgroup will be assessed for non-inferiority. Since the trial was stopped early, the same composite method for the primary outcome and interim analyses will be used.

### Sensitivity analyses

Sensitivity analyses will be performed to determine the following: (a) the effect of high-volume arthroplasty sites; (b) sites with high and low overall registration rates; (c) sites that required multiple compliance audits; and (d) the effect of patients who take long-term aspirin therapy on the results of the analyses for the primary outcome in population 1. The same methods for the main analyses will be used.

### Order of planned analyses

Analyses will be performed in the following order:

- Interim analyses of population 1
- Primary and secondary outcomes for population 1
- Subgroup analyses for population 1
- Primary and secondary outcomes for populations 2, 3 and 4
- Subgroup analyses of population 3
- Sensitivity analyses in population 1

## Additional analyses

### Mortality analysis

In addition to analysing between-group mortality for populations 1, 2, 3 and 4, the between-group 90-day mortality will be analysed for two further populations:

1. All patients undergoing HA or KA over the duration of the study at participating hospitals, regardless of whether they were registered (total population described above, population 5)
2. All patients undergoing elective THA or TKA over the duration of the study at participating hospitals regardless of whether they were registered (a subset of population 5)

Analysing these additional populations will assess the effect of implementing the VTE prophylaxis protocol on mortality at an institutional/departmental level (the unit of randomisation), on an intention-to-treat basis.

### Sub-studies

Data from this trial will be used to form the basis of sub-studies. These will include a sub-study comparing rates of persistent wound drainage between LMWH and aspirin groups at two participating sites and a sub-study investigating rates of post-hospital discharge compliance to either study drug.

## Conclusions

CRISTAL aims to provide much needed definitive evidence about the effectiveness and safety of aspirin compared to LMWH in preventing symptomatic VTE following HA or KA. This statistical analysis plan details the study’s planned analyses, including modifications to intended analyses to account for the early stopping of the trial.

## Data Availability

The datasets during and/or analysed during the current study will be made available from the corresponding author on reasonable request.
